# Geochemical and mineralogical characteristics of shales from the early to middle Permian Dohol Formation in Peninsular Malaysia: Implications for organic matter enrichment, provenance, tectonic setting, palaeoweathering and paleoclimate

**DOI:** 10.1016/j.heliyon.2024.e27553

**Published:** 2024-03-12

**Authors:** Alidu Rashid, Numair Ahmed Siddiqui, Nisar Ahmed, Ali Wahid, Muhammad Jamil, Abdul Aziz Sankoh, John Oluwadamilola Olutoki

**Affiliations:** a Department of Geoscience, Universiti Teknologi PETRONAS, 32610, Seri Iskandar, Perak, Malaysia; b Institute of Geology, University of Azad Jammu and Kashmir, Muzaffarabad, AJK, Pakistan; c Department of Earth Sciences, COMSATS University Islamabad, Abbottabad Campus, Abbottabad, Pakistan; d Department of Civil and Environmental Engineering, University of Energy and Natural Resources, P.O. Box 214, Sunyani, Ghana; e College of Engineering and Computing, George Mason University, 4400 University Dr, Fairfax, VA, 22030, USA

**Keywords:** Inductively coupled plasma mass spectrometry (ICPMS) analysis, Xray fluorescence (XRF) analysis, Major and trace elements, Scanning electron microscopy (SEM), Rare earth elements, Paleoenvironment

## Abstract

The early to middle Permian Dohol Formation is characterized by a significant presence of shale deposits. While these shales exhibit a low potential to generate hydrocarbons, there is a need to ascertain the possible reasons for the low hydrocarbon generation potential. Also, there are several unidentified properties and attributes associated with these shales in terms of their inorganic geochemical characteristics and their mineralogy. This study is focused on using XRF, ICPMS, and SEM with EDX to determine the mineralogical and geochemical characteristics of these shales and use these data to discuss their provenance history and tectonic setting and interpret the paleoclimatic and paleoweathering conditions. The inorganic geochemical analysis shows that the shales from the Dohol Formation are from a felsic igneous source. The shales were also identified to be from a passive margin based on the bivariate plot of SiO_2_ vs log (K_2_O/Na_2_O) and several multidimensional diagram plots. The CIA and CIW data, as well as the A–CN–K plot, all point to a significant degree of chemical weathering, ranging from mild to intense. The Sr/Cu ratio and C-value, combined with various other geochemical proxies, indicate that the shales were formed in warm-humid climatic conditions. The SEM analysis shows that the samples are mainly composed of kaolinite and illite, and this result was supported by the EDX elemental composition. The high terrigenous influx of sediments, the oxic to sub-oxic conditions in which the sediments were deposited, and finally low marine productivity were found to be the reasons for the low TOC in the shales from the Dohol Formation.

## 1 Introduction

Approximately 25% of the sedimentary terrain in Peninsular Malaysia is comprised of black shales from the Paleozoic era [1]. The Dohol Formation is classified as one of the 19 Paleozoic formations found in Malaysia that are characterised by the presence of black shales [1]. The Dohol Formation mostly consists of carbonaceous shale, siltstone, slate, phyllite, and schists [2,3]. Additionally, there are minor occurrences of arenite, limestone, and volcanic deposits within the formation [2]. The deposition of the carbonate unit within the formation, known as the “Sumalayang limestone," is hypothesised to have occurred in an inner neritic shelf setting during the Middle Permian era. This environment was characterised by the presence of fusulinids [4]. The presence of fusulinids within the Sumalayang Limestone Member suggests that this geological formation can be attributed to the late Early Permian to the early Middle Permian period [5]. The shales originating from the Dohol Formation exhibit little hydrocarbon generation potential and are hypothesised to have originated from a deep marine setting [6].

While certain aspects of the Dohol Formation, such as the physical attributes of the rocks and the source rock potential, have been established, it is imperative to ascertain the inorganic geochemical properties of the shales. According to Rashid et al. [6], the shales from the Dohol Formation have low hydrocarbon generation potential due to the low TOC content and the already matured nature of the organic matter. Hence, it is imperative to identify the potential causes for this phenomenon. This can be accomplished by identifying the factors that controlled the deposition of the organic matter. Furthermore, the understanding of the inorganic geochemical characteristics of shales, particularly regarding the major oxide composition, trace elements, and rare earth elements, is currently lacking. This knowledge would be valuable in determining the provenance, tectonic setting, paleoweathering, and paleoclimate conditions.

Provenance studies entail determining the lithologic origins of sediments and/or sedimentary rocks [7]. Provenance refers to all features of a sedimentary rock's ultimate source, including its composition, location, climate, and relief [8]. Trace elements such as Th, Zr, Hf, Nb, Sc, Y, and Cr, together with rare earth elements (REEs) including La, Ce, Nd, Gd, and Yb, are highly suitable for discerning the origin and tectonic setting. This is mostly due to their limited mobility throughout sedimentary processes and brief duration in seawater [9]. Various geochemical proxies, which rely on the analysis of major, trace, and REEs, have the capability to differentiate between distinct tectonic settings of the source terrane [10,11]. Major elements and specific immobile trace elements, such as Th, Sc, Co, Cr, Zr, Hf, and Y, as well as REEs, are useful indicators of the source of rocks and the tectonic setting of clay deposits from various geological contexts [[12], [13], [14]]. The assessment of the extent of weathering exhibited by specific minerals, such as feldspar, may serve as an indicator of the prevailing paleoclimatic circumstances that were present during the sediment's formation [15]. The degree of weathering may vary depending on the prevailing environmental conditions or the distance covered during sediment transportation, resulting in varying levels of intensity, ranging from negligible to moderate to intense [16,17]. The geochemical and mineralogical composition of siliciclastic rocks is significantly influenced by both chemical and physical weathering processes [18,19]. When dissociated from hypergene processes, the information included within paleoweathering profiles presents an advantageous prospect for the reconstruction of paleoclimate [20].

The objective of this study is to determine the factors controlling the organic matter enrichment, to establish the origin and potential source of the shales, to predict the tectonic evolution of the shales, to examine the paleoweathering, and finally to determine the paleoclimate conditions in which the shales were formed. To achieve the aim of this study, inductively coupled plasma mass spectrometry (ICPMS), x-ray fluorescence (XRF) and scanning electron microscopy (SEM) with energy dispersion x-ray (EDX) were employed to determine the inorganic geochemical characteristics and mineralogical composition of the shales.

## 2 Geological setting and the study area

**Fig. 1:**
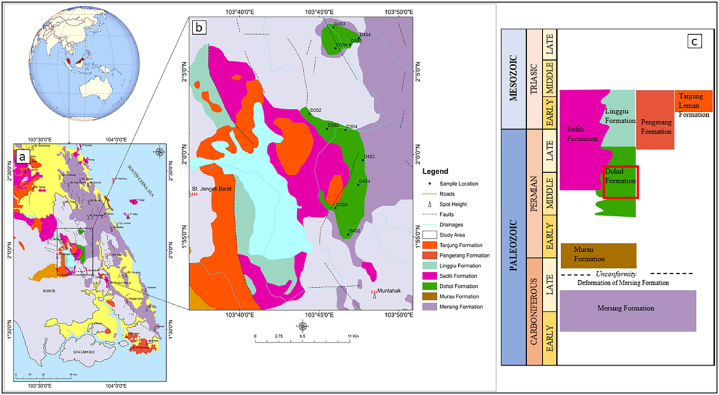
(a) Geological map of the East Johor (b) Map of study area displaying the outcrop locations (c) Stratigraphy of the East Johor region with several Paleozoic formations. Modified after Rashid [3].

**Fig. 2:**
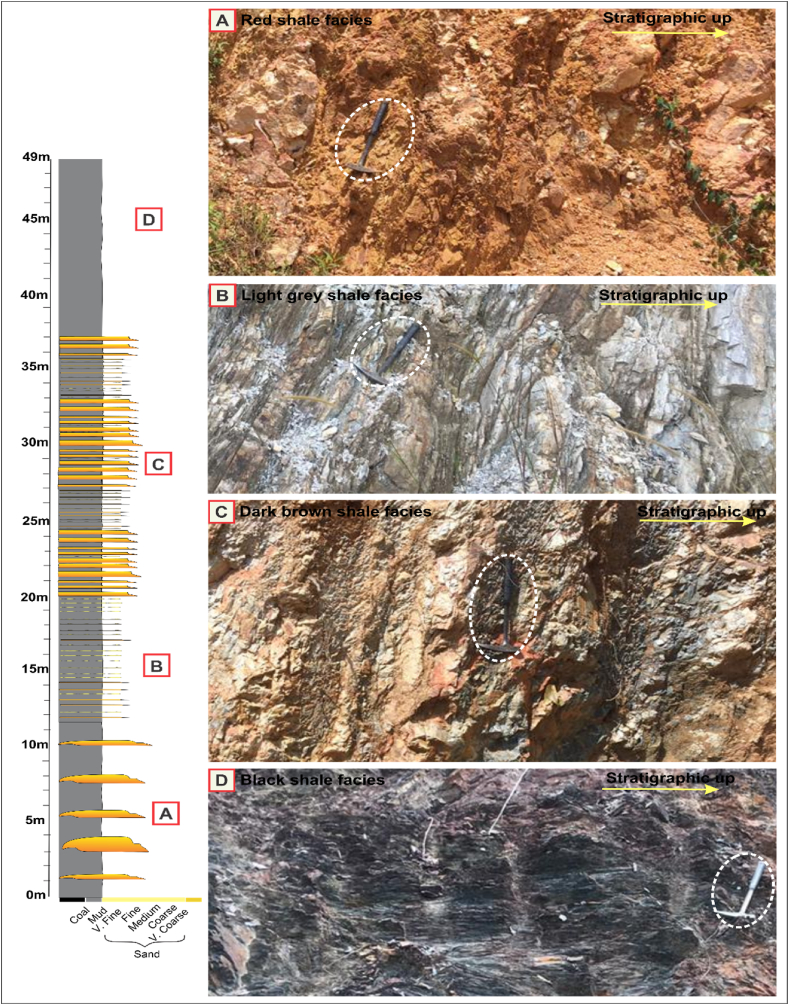
A simplified lithologic log of the shales from the Dohol Formation; (a) Red shale facies (b) Light grey shale facies (c) Dark brown shale facies (d) Black shale facies.

Peninsular Malaysia is approximately 130,265 km^2^ in size. Its greatest length is 750 km and its maximum width is 330 km. Peninsular Malaysia is composed of three geological belts that exhibit a north-south (NAS) trend, as determined by analysis of stratigraphy, structure, magmatism, and geophysical features [25,26]. The region under consideration is geographically demarcated by the Johor Strait in the south, which serves as a natural boundary between the area and Singapore Island, and the Malacca Strait in the west, which separates it from Sumatra Island [21,22]. Peninsular Malaysia is geologically characterised by the presence of the South-East Asian section of the Eurasian Plate, also referred to as Sunderland [22]. The Peninsula is widely recognised for its tectonic stability, characterised by few occurrences of uplifts, tilting, fault movements, and modest down warps [23,24]. Peninsular Malaysia is geographically divided into three distinct belts, namely the Eastern, Western, and Central belts. The classification was established on the many geological attributes exhibited by the belts [25,26]. The Dohol Formation is situated on the Eastern Belt of Peninsular Malaysia. The belt mostly consists of geological formations dating from the Carboniferous to Permian periods. These strata extend from the eastern region of Kelantan to the southern region of Johor, traversing through Terengganu and eastern Pahang [22]. It is postulated that Late Palaeozoic (Permian) witnessed regional metamorphism, folding, and uplift, afterwards succeeded by the sedimentation of an earlier suite of continental deposits, encompassing the Murau and Redang conglomerates [27]. The Eastern belt experienced uplift during the Pan Peninsula Triassic orogeny [27]. Subsequently, a sequence of relatively inclined continental sediments was deposited, presumably uplifted during the late Cretaceous epoch [27]. The potential uplift of the Eastern belt at the end of the Permian era, coinciding with the deposition of Lower Triassic ignimbrite, could explain the lack of younger marine deposits in the area [22]. The Dohol Formation represents a significant geological unit within the region of East Johor, characterised by a diverse assemblage of metasedimentary, metamorphic, volcanic, and siliciclastic rocks of Palaeozoic age [28] (Fig. 1a). The age of Paleozoic rocks is commonly associated with the Carboniferous and Permian periods. Kota Tinggi, Kluang, Jemaluang, and the surrounding areas of Mersing encompassed significant locations where the outcrops of the Dohol Formation are found (Fig. 1b). The Palaeozoic rocks found in East Johor can be categorised into seven lithostratigraphic units, namely Mersing, Marau, Dohol, Linngui, Sedili, Pengerang, and Tanjung Leman [28] (Fig. 1c). Four (4) different shale facies were observed in the Dohol Formation. The facies are red shale, light grey, dark brown and black shale facies. The red shales are sandy, fissile, highly weathered, and overlain by pebbly sands (Fig. 2a). The light grey shales are laminated, and they are seen to have intrusions of quartz veins (Fig. 2b). The dark brown shales are fissile, flaky, and intensely weathered compared to the red shales (Fig. 2c). Finally, the black shales are laminated, with both hard and fissile parts (Fig. 2d). According to Rashid et al. [6], Xray diffraction (XRD) analysis shows that the shale samples consist of mainly kaolinite, illite, and quartz.

## 3 Materials and methods

### 3.1 Rock sampling

A total of 12 shale samples were obtained using a hammer. The hand lens, hammer, GPS, and camera are among the equipment used to identify the samples, pick the locations, and record the outcrop locations. The rock samples were collected at 1 m intervals depending on the size of the outcrop. The initial sample preparation such as crushing, and cleaning was done at Universiti Teknologi PETRONAS geology laboratory.

### 3.2 ICPMS analysis

ICPMS analysis was performed at Universiti Teknologi Malaysia on the twelve (12) shale samples to determine the trace and REEs. The trace and rare earth elements were detected using a Thermo Fisher Scientific iCAP Q ICP-MS by digesting approximately 50 mg of powdered material with 4 ml of a 3:1 combination of 22 M HF and 14 M HNO_3_ in Teflon® vials by heating the solutions at approximately 110 C on a hot plate for 24 h. After complete evaporation, the digestion process was performed twice. The final solutions were made with regards to the internal standard in 2% HNO_3_ solution. The test precision was guaranteed by standardization according to Schudel [29], and several duplicate analyses were done to ensure accuracy.

### 3.3 XRF analysis

The XRF analysis was done using a Philips PW 2400 X-ray spectrometer to detect major oxides like SiO_2_, Na_2_O, Al_2_O_3_, CaO, K_2_O, MnO, MgO, Fe_2_O_3_, TiO_2_ and P_2_O_5_. The current through the tube was 60 mA, while the voltage was 40 kV. To calculate the loss on ignition, powder samples were heated to 1000 °C for 6 h (Loss on ingnition; LOI). The XRF analysis was conducted by the Centralized Analytical Laboratory (CAL) at Universiti Teknologi PETRONAS.

### 3.4 SEM with EDX analysis

The CAL at Universiti Teknologi PETRONAS conducted the SEM with EDX study. The exterior appearance (texture), mineral chemical composition, crystalline structure, and orientation of materials were analysed using SEM. Moreover, using SEM in conjunction with EDX, the chemical compositions of specific regions in the produced samples were determined. For this purpose, 4 samples measuring roughly 70 mm in diameter and 50 mm in height were prepared. In order to eradicate moisture, samples were dried in a 35 °C oven overnight. Following the removal of moisture, the samples underwent a process of carbon coating, which served the purpose of preventing charge accumulation and facilitating the production of a visually distinct image of the target material.

## 4 Results

### 4.1 Major elements

**Fig. 3:**
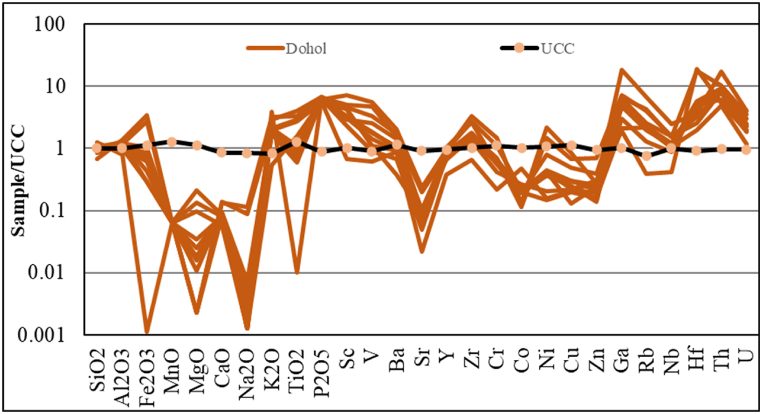
UCC normalized pattern of the shales from the Dohol Formation. Data was normalized to UCC according to Taylor and McLennan [9] and McLennan [82].

The SiO_2_ values are the highest among all the major elements detected. The values range from 45.1% to 81.4% with an average of 66.19%. Al_2_O_3_ and K_2_O are the next highest values detected with average values of 16.88% and 7.41% respectively. The MnO, Na_2_O and MgO have the least values with 0.01%, 0.08% and 0.11% respectively as shown in Table 1. The abundance of SiO_2_ demonstrates a high concentration of quartz minerals. High alumina contents are commonly recorded in argillaceous and clayey sediments [30]. The high levels of K_2_O are indicative of an increasing proportion of illite or K-feldspar [31]. The values for SiO_2_, Fe_2_O_3_, MnO, MgO, CaO and Na_2_O are lower than the Upper Continental Crust (UCC) values according to Taylor and McLennan [9] (Fig. 3). Whilst Al_2_O_3_, K_2_O, TiO_2_ and P_2_O_5_ have values higher than the standard UCC values according to Taylor and McLennan [9].

**Table 1:** Some major oxide concentrations (%) of shale samples from the Dohol Formation.

Sample number	Formation	SiO_2_	Al_2_O_3_	Fe_2_O_3_	MnO	MgO	CaO	Na_2_O	K_2_O	TiO_2_	P_2_O_5_
S1	Dohol	45.10	20.80	15.60	0.01	0.01	0.56	0.45	10.90	1.65	1.15
S2	Dohol	56.40	17.90	12.10	0.01	0.01	0.59	0.34	7.14	1.44	1.11
S3	Dohol	57.40	16.40	3.80	0.01	0.30	0.34	0.01	13.30	0.01	1.04
S4	Dohol	81.40	12.10	0.01	0.01	0.01	0.34	0.01	1.83	0.79	0.97
S5	Dohol	69.40	18.80	1.31	0.01	0.22	0.25	0.01	6.74	1.37	0.94
S6	Dohol	64.00	17.10	2.91	0.01	0.47	0.36	0.01	9.85	2.02	1.16
S7	Dohol	70.10	13.60	3.50	0.01	0.04	0.39	0.02	7.08	0.54	1.06
S8	Dohol	68.39	16.08	2.74	0.01	0.04	0.36	0.01	5.85	0.30	1.01
S9	Dohol	65.80	18.20	4.63	0.01	0.08	0.33	0.01	6.37	0.35	1.03
S10	Dohol	67.00	19.81	1.96	0.01	0.02	0.39	0.03	6.63	0.42	0.98
S11	Dohol	73.79	15.38	1.30	0.01	0.04	0.38	0.02	6.83	0.48	1.05
S12	Dohol	75.47	16.41	3.23	0.01	0.05	0.36	0.01	6.46	0.42	1.09
	Min	45.10	12.10	0.01	0.01	0.01	0.25	0.01	1.83	0.01	0.94
	Max	81.40	20.80	15.60	0.01	0.47	0.59	0.45	13.30	2.02	1.16
	Average	66.19	16.88	4.42	0.01	0.11	0.39	0.08	7.41	0.81	1.05

### 4.2 Trace elements

Among the trace elements detected Ba has the highest value of 628.16 ppm followed by Zr and Rb with values of 350.51 and 300.03 ppm respectively. Co and Cu have the lowest values of 4.26 and 8.67 ppm respectively (Table 2). The average values of all the elements are higher than both the UCC and Post Archean Australian Shale (PAAS) standards except a few elements like Co, Cu, Y, Sr, Zn, Nb (Fig. 3).

**Table 2:** Trace element composition (ppm) of shale samples from the Dohol Formation.

Sample number	Formation	Ba	Ni	Zr	Cr	Cu	V	Rb	Ga	Sr	Hf	Nb	U	Sc	Co
S1	Dohol	967.54	18.33	519.89	90.92	3.30	350.99	472.57	109.64	81.45	101.82	18.50	9.96	62.59	3.59
S2	Dohol	871.26	65.48	307.60	59.07	16.91	505.91	450.69	123.95	69.64	14.95	18.62	5.42	68.12	1.94
S3	Dohol	1135.18	95.41	623.43	123.20	21.46	593.14	758.92	307.28	80.89	20.99	29.19	11.07	96.50	2.24
S4	Dohol	489.06	6.99	123.82	18.35	5.20	64.89	43.66	32.78	7.76	111.22	4.97	5.30	9.29	8.18
S5	Dohol	519.78	16.01	493.97	45.72	6.25	118.60	285.07	108.26	21.70	18.62	18.61	8.31	58.51	3.27
S6	Dohol	208.34	6.47	274.45	47.36	4.92	119.34	240.86	35.87	30.67	11.47	12.99	3.28	57.98	3.36
S7	Dohol	375.54	8.98	256.05	34.12	5.43	97.20	144.19	50.30	17.29	28.75	10.63	5.25	64.21	4.48
S8	Dohol	567.82	18.51	330.26	51.00	7.24	191.38	254.87	82.83	32.81	29.99	14.40	6.43	50.46	3.49
S9	Dohol	784.98	35.21	287.42	51.11	12.36	269.03	246.26	107.68	35.23	32.68	13.93	6.83	79.56	3.29
S10	Dohol	495.17	16.21	319.84	47.04	7.65	152.77	218.40	79.08	25.42	26.57	13.68	7.23	80.34	3.77
S11	Dohol	578.54	19.48	324.66	50.60	7.68	193.60	250.11	84.64	32.21	29.88	14.28	6.54	61.98	3.49
S12	Dohol	544.67	18.62	344.78	54.52	5.67	196.43	234.78	88.32	36.65	28.48	12.36	7.89	59.88	3.49
	Min	208.34	6.47	123.82	18.35	3.30	64.89	43.66	32.78	7.76	11.47	4.97	3.28	9.29	1.94
	Max	1135.18	95.41	623.43	123.20	21.46	593.14	758.92	307.28	81.45	111.22	29.19	11.07	96.50	8.18
	Average	628.16	27.14	350.51	56.08	8.67	237.77	300.03	100.89	39.31	37.95	15.18	6.96	62.45	3.72

### 4.3 REEs

**Fig. 4:**
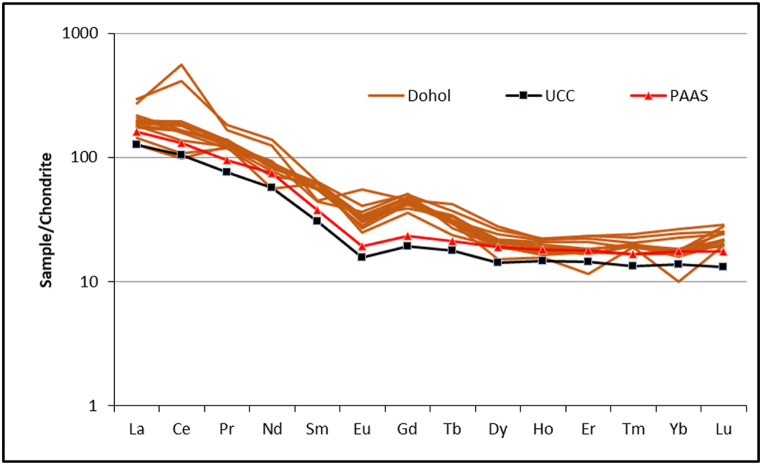
REE trends in shales from the Dohol Formation. The data was normalized to UCC and PAAS according to Taylor and McLennan [9].

The rare earth elements (REEs) with lower atomic numbers (La–Sm) are called Light REEs (LREE), whereas those with higher atomic numbers are called “heavy" REEs (HREE) (Gd – Lu). The Heavy REEs (HREEs) have very high values of all the REE analysed while the LREE have relatively low values. Ce has the highest value of 129.16 ppm followed by La and Nd with values of 50.615 and 41.04 ppm respectively. The lowest REE values are from Tm, Lu, and Ho with values of 0.49, 0.54 and 1.02 ppm respectively (Table 3). The average values of all the elements are higher than both the UCC and PAAS standards as shown by the chondrite normalized REE pattern for the shales (Fig. 4). The studied samples show a wide variation in the elements according to the chondrite normalized REE pattern. The chondrite normalized REE pattern is showing the following characteristics: (a) LREE enrichment (La/Yb_CN_ = 7.8–14.2 _CN_ - Chondrite-normalized), (b) HREE deletion (Sm/Yb_CN_ = 2.0–6.2), and (c) negative Eu anomalies (Eu/Eu* average = 0.68).

**Table 3:** REE concentrations (ppm) of shale samples from the Dohol Formation.

Sample number	Formation	La	Ce	Pr	Nd	Sm	Eu	Gd	Tb	Dy	Ho	Er	Tm	Yb	Lu	Th
S1	Dohol	64.10	340.90	15.33	57.01	6.62	3.09	9.00	1.51	6.82	1.20	3.57	0.51	3.61	0.59	108.08
S2	Dohol	52.05	101.73	11.71	42.83	6.48	2.04	10.07	0.97	4.71	0.89	2.75	0.41	2.88	0.47	88.49
S3	Dohol	49.31	97.70	11.06	40.31	9.21	1.93	9.46	1.12	5.92	1.17	3.72	0.59	4.28	0.70	186.62
S4	Dohol	30.11	59.97	11.43	25.55	9.11	1.51	8.37	1.07	3.73	0.85	1.84	0.47	1.61	0.48	56.78
S5	Dohol	70.20	254.43	16.87	63.31	9.45	2.30	9.97	1.34	6.44	1.21	3.73	0.56	3.92	0.62	93.26
S6	Dohol	33.87	65.95	11.11	32.04	9.47	1.40	7.14	0.86	4.64	0.93	2.79	0.41	2.68	0.52	51.75
S7	Dohol	41.52	100.21	12.89	37.28	9.34	1.69	8.41	1.07	4.81	0.99	2.68	0.48	2.57	0.50	64.95
S8	Dohol	46.76	119.19	12.75	40.86	8.12	1.58	8.86	1.12	5.19	1.09	2.93	0.49	2.95	0.53	84.89
S9	Dohol	42.59	84.16	11.40	35.33	8.16	1.81	9.27	1.05	4.70	0.96	2.66	0.48	2.71	0.54	97.88
S10	Dohol	43.35	99.58	12.41	38.02	9.31	1.75	8.66	1.08	5.07	1.03	2.91	0.50	2.69	0.62	84.56
S11	Dohol	45.98	113.06	12.57	39.98	8.48	1.93	8.21	1.23	5.13	1.02	2.90	0.49	2.92	0.60	58.23
S12	Dohol	44.47	110.48	11.38	41.22	8.97	1.89	7.78	1.19	5.38	1.14	3.32	0.46	2.87	0.68	83.07
	Min	30.11	59.97	11.06	25.55	6.48	1.40	7.14	0.86	3.73	0.85	1.84	0.41	1.61	0.47	51.75
	Max	70.20	340.90	16.87	63.31	9.47	3.09	10.07	1.51	6.82	1.21	3.73	0.59	4.28	0.70	186.62
	Average	47.03	128.95	12.58	41.15	8.56	1.91	8.77	1.13	5.21	1.04	2.98	0.49	2.97	0.57	88.21

### 4.4 Mineral composition

**Fig. 5:**
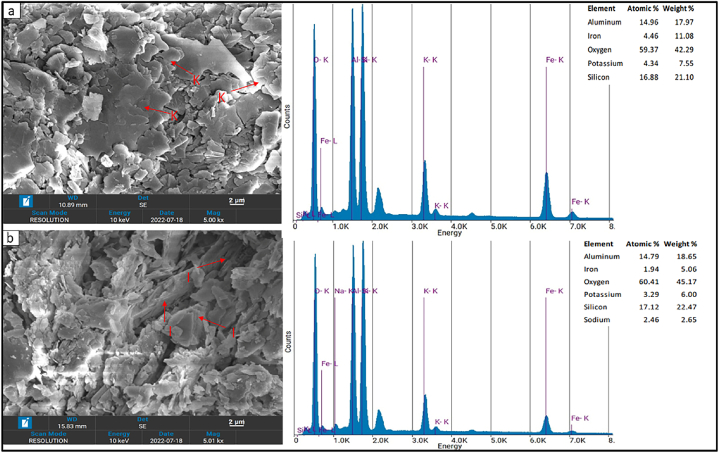
SEM image with EDX report of shale samples from the Dohol Formation; (a) showing the presence of K-Kaolinite (b) showing the presence of I-Illite.

The SEM images obtained show that the samples from the Dohol formation are high in clay minerals, quartz, and feldspars. The clays minerals observed are mainly kaolinite and illite. The kaolinite was identified as it has platy or flaky structures that are thin and elongated in shape [32,33] (Fig. 5a). Occasionally, the particles may display a slightly rough or rippled texture due to variations in the thickness of the layers. The illite is somewhat like the kaolinite in terms of its flaky and platy nature however they are arranged in aggregates and clusters [34] (Fig. 5b). The quartz shows prism-like features which often exhibit twinning. Lastly the feldspars show intergrown crystals which are typically rough and irregular. According to the EDX report, there is a significant presence of aluminium, silicon, and oxygen in the analysed shale samples (Fig. 5a&b). These elements are often found in minerals such as kaolinite and illite, which the SEM examination confirmed were present in the shales.

## 5 Discussion

### 5.1 Provenance

**Fig. 6:**
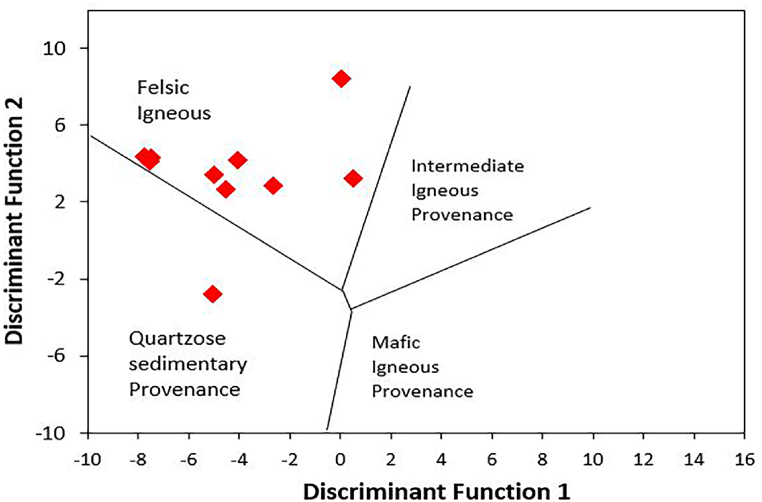
Discriminant plot suggested by Roser and Korsch [39] using discriminant function 1 and discriminant function 2 which interprets provenance. The discriminant function 1 and 2 were calculated using the following formulae: Discriminant Function 1 = −1.773 TiO_2_ + 0.607 Al_2_O_3_ + 0.76 Fe_2_O_3_ - 1.5 MgO +0.616 CaO +0.509 Na_2_O – 1.224 K_2_O – 9.09. Discriminant Function 2 = 0.445TiO_2_ + 0.07 Al_2_O_3_ – 0.25 Fe_2_O_3_ - 1.142 MgO +0.438 CaO +1.475 Na_2_O +1.426 K_2_O – 6.861.

**Fig. 7:**
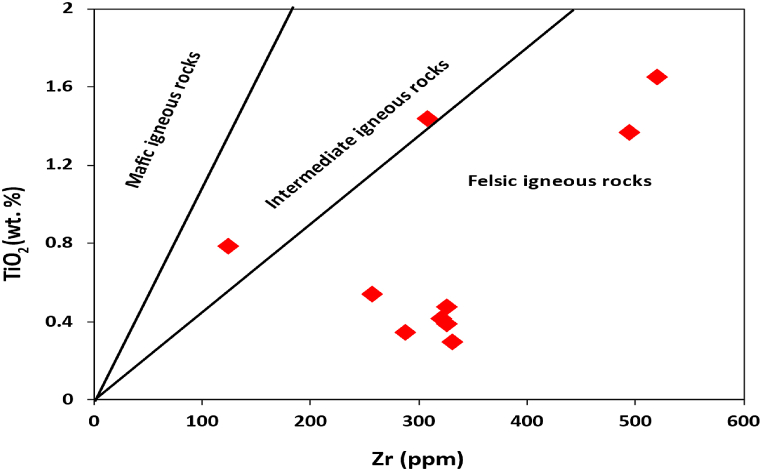
Discrimination plot of TiO_2_ vs Zr for shales from Dohol Formation after Hayashi et al. [43].

**Fig. 8:**
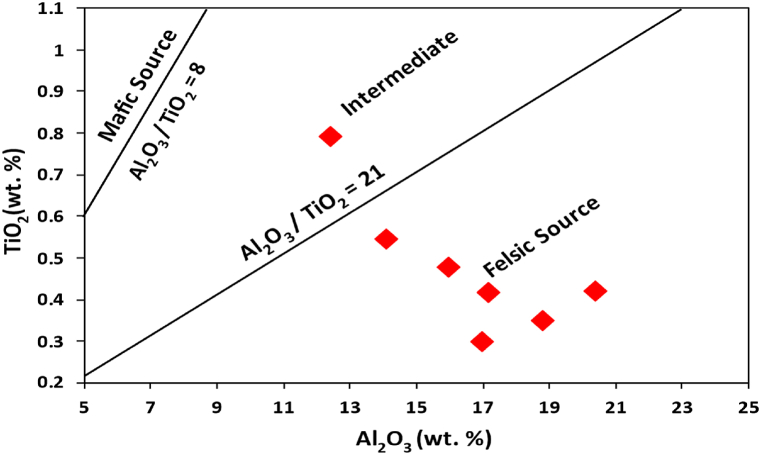
Discrimination diagram of Al_2_O_3_ versus TiO_2_ diagram after McLennan et al. [44] showing intermediate to felsic source.

Clastic sediment geochemistry has been widely employed to determine the provenance of rocks. Argillaceous rocks are important for provenance study due to their homogeneity and low permeability after deposition [13,[35], [36], [37]]. Furthermore, depositional, or post-depositional sedimentary processes such diagenesis, weathering, and metamorphism do not significantly affect all elements. Since REE and a small number of immobile elements (such as Cr, Th, Sc, and Co) are included in that resistant list, they can be utilised as provenance markers [38]. According to the major oxide-based provenance discriminant plot by Roser and Korsch [39], the shales from the Dohol Formation are from a felsic igneous provenance region (Fig. 6). This shows that most of these shales are originally derived from felsic rocks which have undergone sedimentation to form these shales. In general, the relative enrichment of elements such as Sc and Co, which are typically incompatible and less abundant in compactible materials, suggests a felsic provenance and a history of significant weathering [40]. The concentrations of immobile elements, namely La and Th, are comparatively higher in rocks that have originated from felsic sources. Conversely, mafic rocks tend to have a higher concentration of Sc and Co in comparison to felsic rocks [40]. This finding provides further evidence that the shale samples obtained from the analysed formations originate from a felsic origin (Table 2). The ratios of compatible and incompatible REEs are also crucial in determining whether the parent rock is felsic or mafic. Mafic rocks have low LREE/HREE ratios and no Eu anomalies, whereas felsic rocks have comparatively high LREE/HREE ratios [41,42]. As a result, the generally high values of the high LREE/HREE values indicates a felsic origin. Zr concentrations are often utilised to describe the type and composition of source-area rocks. When the shales show strong positive correlation between Zr and TiO_2_ (r = >0.5), it suggests that the concentration of certain accessory minerals such as zircon, monazite, rutile and ilmenite could be higher. Average zircon content of the Dohol shales varies from 123 to 623 ppm which is above the PAAS (Zr = 210) (Table 2) and higher than in the Proterozoic Shales (Zr = 151) [7]. A TiO_2_ vs Zr plot distinguishes three different source-area rock types, i.e., felsic, intermediate, and mafic. The TiO_2_ vs Zr plot of the Dohol shales (Fig. 7) shows a predominantly felsic igneous rocks and partially intermediate source-area rocks [43]. This also confirms the interpretations from the Al_2_O_3_/TiO_2_ ratio. The Al_2_O_3_/TiO_2_ ratios in clastic rocks are commonly employed to infer the composition of the source-area rocks. This is because the Al_2_O_3_/TiO_2_ ratio tends to increase from 3 to 8 for mafic igneous rocks, from 8 to 21 for intermediate rocks, and from 21 to 70 for felsic igneous rocks [44]. The Al_2_O_3_/TiO_2_ ratios of the Dohol Formation shales range from 8 to 54 (Appendix Table A1) corresponding to a source area made up of intermediate to felsic granitoid rocks (Fig. 8).

### 5.2 Tectonic setting

**Fig. 9:**
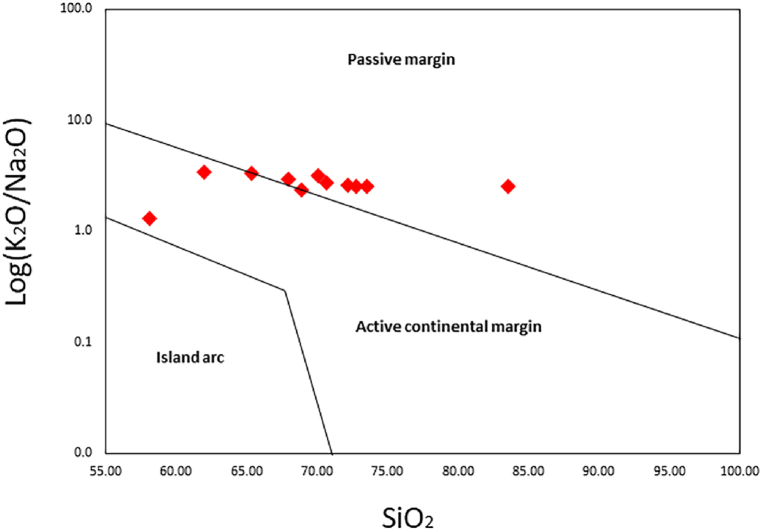
The bivariate plot of SiO_2_ vs log (K_2_O/Na_2_O) interpretating the tectonic setting of the shales after Roser and Korsch [83].

**Fig. 10:**
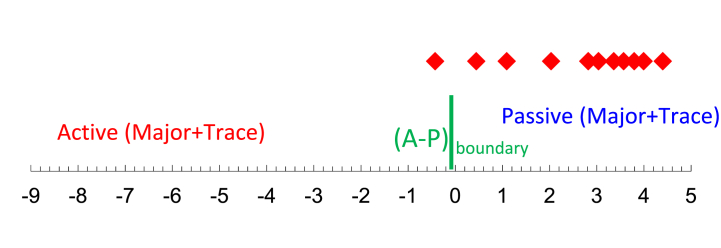
Major element (M) multidimensional discriminant function diagram distinguishing tectonic settings of shale formations according to Verma and Armstrong-Altrin [46].

To investigate and infer the tectonic settings of ancient sedimentary basins, numerous scientists have extensively employed the major and trace element concentrations of sediments [[9], [10], [11],^45^]. Trace elements are more useful than major elements for differentiating tectonic settings of siliciclastic due to the concentration of accessory phases, which may be sensitive to hydraulic concentration [11]. In this study, we employed all available geochemical data (major, trace, and REE) to deduce the tectonic settings of the studied area. The bivariate plot of SiO_2_ vs log (K_2_O/Na_2_O) was employed to interpret the tectonic settings of the shales. Most of the samples from the Dohol Formation are falling in the passive margin (Fig. 9), whereas the others are more towards the active continental margin. The multidimensional diagram from Verma and Armstrong-Altrin [46] was also used to distinguish between active and passive margin settings. According to their classification, the shales from the Dohol Formation are from the passive margin which includes rift setting sediments [46] (Fig. 10). Isometric log-ratio transformations of elemental concentrations was employed to calculate novel discriminant functions based on major elements (DF_(A-P)M_) and major and trace elements (DF_(A-P)MT_). The result from these interpretations shows that the shales from the Dohol Formation is from a passive margin.

When plagioclase occurs in high concentrations, it can be linked to the environment and source rock. As reported by Surjono et al. [28], plagioclase displays peculiar mineralogical concentration ranging from 0% to 40%, particularly in Tanjung Leman and the Dohol formations. The same has been found in the XRD analysis of the shales in one of the previously published studies on the Dohol Formation [6]. The Dohol Formation demonstrates the presence of a significant amount of plagioclase, which may have come from any magmatic arc that continuously supplied the depositional basin with plagioclase-rich material ([6]; Table 1). By combining the provenance with the tectonic setting, we can correlate the possible source and geological environment dominated during the deposition of the Dohol Formation. The Dohol Formation was directly affected by the volcanic events which were occurring within the Peninsular Malaysia that possibly occurred within the period of Early to Late Permian (255–300 Ma). It implies that the volcanic materials with high feldspar concentrations (magmatic arc) contributed to the sediment supply. Those volcanic input may have originated from the Sedili Formation, which interfinger with the Dohol Formation as well as with Linggiu sandstones, or they may have come from an earlier volcanism event [28]. Therefore, it can be deduced that the passive margin tectonic setting resulted mainly from the older sediments, whereas some of the shale samples are that are from the active margin tectonic setting resulted from volcanic sources.

As discussed by Surjono et al. [28] the Dohol, Linggiu, and Tanjung Leman formations reflect a significant change in relation to the tectonic setting occurring within the Peninsular Malaysia, particularly during the Early to Late Permian (255–300 Ma). This displays both recycled lithic material from the earlier strata and a volcanic source. The volcanism event that may have taken place between the Early and Late Permian had a direct impact on the Linggiu and Dohol Formations. The occurrence of volcanic clay-sized matrix material changed to sericite ([28]; Fig. 3) can be used to understand the influence of volcanism.

### 5.3 Weathering and sediment recycling

**Fig. 11:**
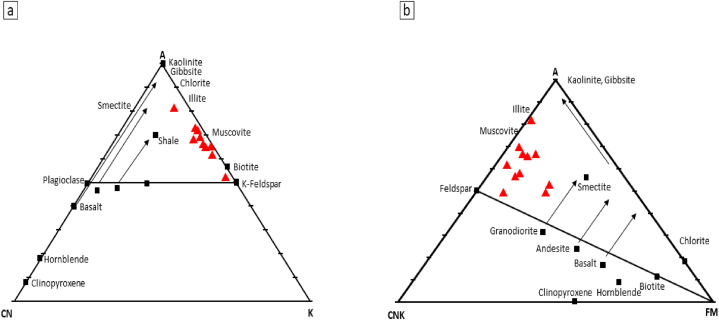
(a) A–CN–K ternary plot after Nesbitt and Young [20] showing the weathering profile of the shales (b) A-CNK-FM plot after McLennan et al. [18] indicating the pattern of weathering.

**Fig. 12:**
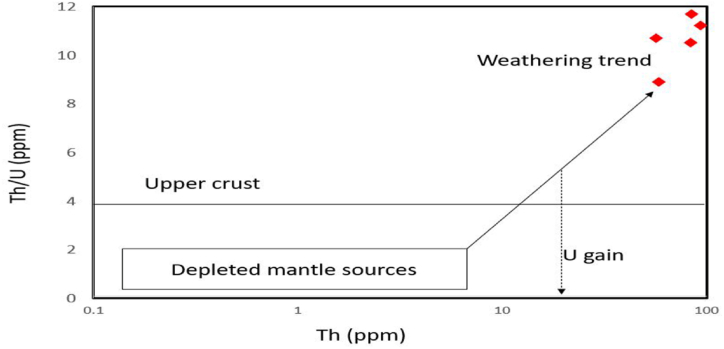
Th/U (ppm) versus Th (ppm) cross-plot for weathering and source behaviour after McLennan et al. [18].

The constituents of siliciclastic rocks are influenced by multiple factors, encompassing the maturity of sediments and the extent of chemical weathering experienced by the parent rock [18,[47], [48], [49]]. During chemical weathering, feldspar transforms into clay minerals, and the gradual increase in weathering leads to removal of easily displaced mobile cations (K^+^, Na^+^ and Ca^2+^) in comparison to more stable elements/residual constituents (Al^3+^ and Ti^4+^) [50]. Fresh igneous rocks that have not undergone weathering have CIA and PIA values between 45 and 55, while heavily weathered sediments that contain a lot of kaolinite, gibbsite, chlorite, and boehmite have values as high as 100. The presence of the clay mineral groups smectite and illite, as well as CIA and PIA values between 60 and 80, indicate moderate weathering [1]. Chemical Index of Alteration (CIA; [20]) and Plagioclase Index of Alteration (PIA; [49]) were used to evaluate the intensity of weathering. CIA is defined as follows: CIA = 100*[Al_2_O_3_/(Al_2_O_3_ + CaO⁎ + Na_2_O + K_2_O)], where the major element concentrations are presented as mol%. The amount of CaO incorporated in the silicate fraction is designated as CaO*. Assuming appropriate values of the Ca/Na ratios of the silicate material, McLennan [40] provided an indirect approach for estimating the CaO concentration of the silicate fraction. To calculate the CaO content of the silicate fraction (CaO*), the molar proportion of P_2_O_5_ must be subtracted from the molar percentage of total CaO. If the remaining number of moles is less than that of Na_2_O (CaO* <Na_2_O), this CaO* value is adopted for calculations. Otherwise, CaO* is assumed to be equivalent to Na_2_O. This approach can yield minimum CIA values because the Ca is lost more quickly during weathering more than the Na. CaO* values were used to calculate the CIA weathering index. In this case, after subtraction, the “remaining number of moles" is found to be smaller than the molar percentage of Na_2_O. The weathering intensity of the shales was assessed using the most widely used chemical indices, including the Chemical Index of Alteration (CIA), the Chemical Index of Weathering (CIW) and the Plagioclase Index of Alteration (PIA). The CIA value may be reduced due to K-metasomatism, signalling that it should be utilised with caution [50,51]. The calculated CIA and PIA values of the Dohol Formation's shales indicate moderate to high weathering (CIA values range from 57 to 98 and PIA from 91 to 107). Only two samples show CIA of 57 and 66 suggesting moderate weathering nature due to high content of iron oxide cement and less clay minerals (Appendix Table 1A). The A–CN–K ternary diagram is a straightforward and reliable method for determining chemical weathering trend and the effects of K-metasomatism, however the values must be transformed from weight percent (%) to molecular fraction [52,53]. The shale samples plotted on the A–CN–K ternary plot after Nesbitt and Young [20] are falling near the A–K border as a result the average source rock compositions and the effects of K-metasomatism cannot be recognised [54]. This behaviour could be due to the consequence of the annihilation of plagioclase and the elimination of CaO and Na_2_O because of continuous weathering and are indicative of intensive weathering (Fig. 11a). The examined samples may contain muscovite as detrital components or as a result of regional metamorphism, resulting in an enrichment of K [38]. When plotted in the A-CNK-FM plot after McLennan et al. [18], the sediments move away from the CNK-FM line, this suggests weathering, while parallel movement indicates sorting (Fig. 11b). The Th/U (ppm) versus Th (ppm) cross-plot after McLennan et al. [18] shows that the shales from the Dohol Formation are derived from weathering of source rocks and/less influenced by recycling (Fig. 12). Moreover, due to the very high values of CIA, if the sediments have experienced K-enrichment, this may reflect a sediment recycling nature and thus a cumulative effect [54]. One of the samples from the studied area shows very high CIA values (up to 97.7), indicating that the sediments underwent a recycling process. Furthermore, the ∑REE abundances (Appendix Table 1A) reflect the recycling process. High ∑REE contents suggest a possible control by differing amounts of accessory minerals (e.g., zircon) and/or quartz due to recycling processes [54]. In this study, ∑REE values range from 136.2 to 484 ppm, with an average of 238 ppm, higher than that of the PAAS (184.77 ppm) and UCC (146.37 ppm), suggesting the recycling of the Dohol sediments.

### 5.4 Paleoclimate conditions

The evaluation of sedimentary rocks' mineralogy, weathering, and chemical composition necessitates the consideration of climate as a crucial factor [55]*.* Generally warm and humid environments are associated with intense chemical weathering, whereas cold and arid conditions are associated with weak chemical weathering [56]. For the study of climatic variations, the C- Value formular from refs. [57,58] was used: C-value = ∑(Fe + Mn + Cr + Ni + V + Co)/∑(Ca + Mg + Sr + Ba + K + Na). Other paleoclimatic indicators used in this work were ratios of elements such as: Sr/Cu, Sr/Ba, Rb/Sr; and Mg/Ca. According to Lerman and Stumm [59], Sr/Cu ratios have an essential role in paleoclimate signals. Sr/Cu ratios greater than 5.0 ppm show hot and arid paleo-weather, and when it is within 1.3–5.0 ppm (<5), implies warm more humid climate. From the analysed samples, except for three samples (Sr/Cu = 24.6, 6.2 and 6.4; hot arid), rest of the samples show a range of Sr/Cu values from 1.49 to 4.5 with an average value of 3.4, suggesting an overall warm and more humid climate (Appendix Table 2A). The Sr/Ba values > 1 and <1 indicate arid and humid climatic conditions, respectively [60]. The Dohol shales have Sr/Ba ratios that range from 0.0.15 to 0.14, and the majority of the samples have values that are less than 1, on average 0.06 reflecting a humid climatic condition at the time of deposition. The Rb/Sr values > 0.5 and <0.5 indicate humid and arid climatic conditions, respectively [61]. The Dohol shales have Rb/Sr ratios that range from 5.62 to 13.13, with an average of 7.84 reflecting a humid climatic condition at the time of deposition. Mg/Ca ratios highly are susceptible to climate change. Arid climates are typically indicated by high Mg/Ca ratios, while humid climates are generally indicated by low ratios [62]. Following the above assumption provided by Yan et. al [62], the shales in the Dohol Formation were formed during humid conditions.

**Fig. 13:**
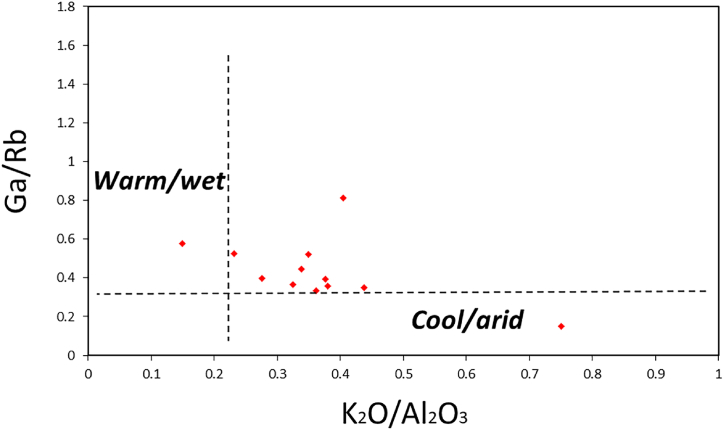
The Ga/Rb vs K_2_O/Al_2_O_3_ plot according to Roy and Roser [63] for interpretation of paleoclimate of rocks.

The Ga/Rb vs K_2_O/Al_2_O_3_ plot according to Roy and Roser [63] was also used to determine the paleoclimate of the shales and it also shows that the samples from the Dohol Formation are away from a cool and dry region and are more toward warm and humid section (Fig. 13a). C- values of <0.4 are characterised by arid climates while C-values of >0.4 denotes semi humid to humid climatic conditions [58,64]. The C-values of the shales in the Dohol Formation range from 0.19 to 0.74 (average = 0.47). Except for three samples (C-values = 0.19, 0.34 and 0.37; arid), the rest of the samples indicate a predominately humid to semi humid paleoclimate. Overall, by combing all the geochemical proxies it can be concluded that the samples from Dohol Formation were deposited in warm and humid paleoclimatic conditions.

### 5.5 Terrigenous influx

**Fig. 14:**
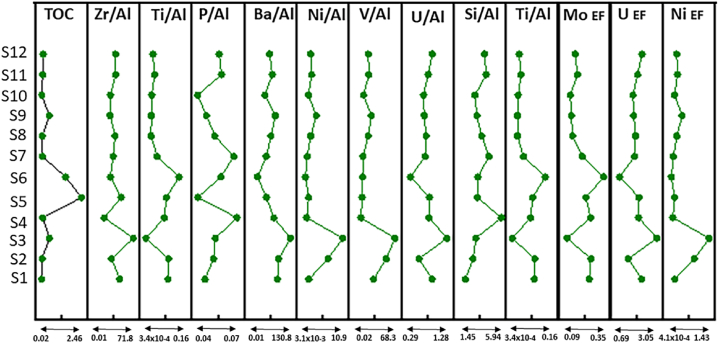
Vertical distribution of terrigenous influx, paleoredox and paleoproductivity proxies.

Aluminium (Al) and Titanium (Ti) possess highly stable chemical characteristics that are never influenced by weathering or diagenesis. As a result, they are frequently employed as proxies for terrestrial influx [65]. The primary source of elemental Al is mostly aluminosilicate clay minerals, while Ti is predominantly found in aluminosilicate and Ti-bearing heavy minerals, such as ilmenite and rutile [66]. The Si/Al, Ti/Al, and Zr/Al ratios can be used as proxies to determine the input of terrigenous sediments [67]. The Si/Al plot of the Dohol Formation exhibits some correlation with TOC, indicating that the detrital input did not significantly influence the organic matter concentration of the rocks from the Dohol Formation (Fig. 14). The Ti/Al plot also shows the Dohol Formation exhibits some correlation with TOC, indicating that the detrital input did not significantly influence the organic matter concentration (Fig. 14).

### 5.6 Paleoredox conditions

Redox-sensitive trace elements (RSTEs), including Mo, U, and V, exhibit various geochemical characteristics in the water column. These elements are well recognised as indicators of redox conditions in both modern and ancient sedimentary environments [[68], [69], [70], [71]]. Under reducing conditions, the sediments can accumulate higher concentrations of U and V. This is because U and V ions in the water column can undergo reduction, transforming into stable chemical compounds that become immobile and are subsequently deposited in the sediments [69]. In sedimentary environments, the U/Th and V/Cr ratios increase as the oxidation degree decreases. This is because Cr is reduced from Cr^4+^ to Cr^3+^ and is then deposited into sediments in oxygen-depleted conditions. On the other hand, Th is often not influenced by changes in redox conditions. The U/Th ratios in all the samples analysed are significantly low, suggesting the presence of oxidized conditions in the bottom waters [72] (Table 4). The U/Al ratios are extremely low which indicates oxidized conditions in the bottom waters however the ratios from the V/Al ratios are high which contradicts with the values from the U/Al ratio (Table 4).

**Table 4:** Elemental ratios of the rocks used for organic matter enrichment analysis.

Sample Number	Formation	TOC	Zr/Al	Ti/Al	P/Al	Ba/Al	Ni/Al	V/Al	U/Al	Si/Al	Ti/Al	Mo EF	U EF	Ni EF	P EF	U/Th	P/Ti
S1	Dohol	0.030	47.235	0.090	0.046	87.907	1.665	31.889	0.905	1.915	0.090	0.230	2.162	0.217	0.001	0.092	0.507
S2	Dohol	0.050	32.475	0.091	0.051	91.984	6.913	53.412	0.572	2.783	0.091	0.242	1.367	0.899	0.001	0.061	0.561
S3	Dohol	0.500	71.839	0.000	0.052	130.809	10.994	68.349	1.276	3.092	0.000	0.089	3.048	1.429	0.001	0.059	151.407
S4	Dohol	0.090	19.338	0.074	0.066	76.382	1.092	10.135	0.828	5.943	0.074	0.238	1.978	0.142	0.001	0.093	0.898
S5	Dohol	2.460	49.654	0.083	0.041	52.249	1.609	11.922	0.835	3.261	0.083	0.210	1.996	0.209	0.001	0.089	0.497
S6	Dohol	1.480	30.331	0.134	0.056	23.025	0.715	13.189	0.362	3.306	0.134	0.323	0.866	0.093	0.001	0.063	0.418
S7	Dohol	0.070	35.579	0.045	0.064	52.183	1.248	13.507	0.729	4.553	0.045	0.186	1.742	0.162	0.001	0.081	1.415
S8	Dohol	0.060	38.825	0.021	0.052	66.753	2.176	22.498	0.756	3.758	0.021	0.124	1.805	0.283	0.001	0.076	2.481
S9	Dohol	0.490	29.845	0.022	0.047	81.508	3.656	27.935	0.709	3.194	0.022	0.116	1.693	0.475	0.001	0.070	2.151
S10	Dohol	0.040	30.519	0.024	0.041	47.248	1.547	14.577	0.690	2.989	0.024	0.110	1.648	0.201	0.001	0.086	1.704
S11	Dohol	0.090	39.898	0.035	0.056	71.099	2.394	23.792	0.804	4.239	0.035	0.155	1.921	0.311	0.001	0.076	1.600
S12	Dohol	0.110	39.702	0.029	0.055	62.719	2.144	22.619	0.909	4.063	0.029	0.142	2.171	0.279	0.001	0.092	1.911

The redox-sensitive elements, such as U, Mo, and V, tend to be less soluble in water with low oxygen levels, leading to an increase in their concentration in sedimentary layers formed under oxygen-depleted conditions. Conversely, these elements are more soluble in oxygen-rich conditions, resulting in lower concentrations in sediments deposited in water columns with high oxygen levels [69,73]. The presence of low molybdenum enrichment factor (MO_EF_) and uranium enrichment factor (U_EF_) values suggests that the rocks were formed in oxic conditions, which aligns with the findings of other analyses [74] (Table 4). Despite certain inconsistencies in the paleoredox conditions of the Dohol Formation, most of the evidence suggests that the shales were formed in an environment characterised by an oxic-suboxic conditions.

### 5.7 Paleoproductivity

Phosphorus (P), barium (Ba), copper (Cu), and nickel (Ni) are commonly used as major productivity markers to assess biological metabolic activities in marine habitats [75]. Low total organic carbon (TOC) content is typically associated with photosynthetic primary paleoproductivity and the rate at which organic matter is deposited during burial and remineralization [76,77]. The concentration of Cu, Pb, Zn, Ni, Cd, Mo, and V is significantly higher in areas with high levels of organic carbon. These metals are attracted to and attached to terrestrial organic matter through either electrostatic forces or oxygen-containing functional groups. They are then transported by the fluvial system and eventually deposited in sediments. The Cu/Al values of the Dohol Formation is very low which indicates that there was low marine productivity during the formation of the rocks [78,79] (Table 4). This could be because of reduced nutrient availability, decreased light penetration, or other factors that limit diatom growth. Also, the enrichments of P (P _EF_) have very low values, and this also proves that there was low marine productivity in the shales (Table 4). The P/Ti ratio is also used to predict marine productivity. However, in this case the samples have low P/Ti values which shows the shales were formed in an environment with moderate to low marine productivity.

### 5.8 OM accumulation model

**Fig. 15:**
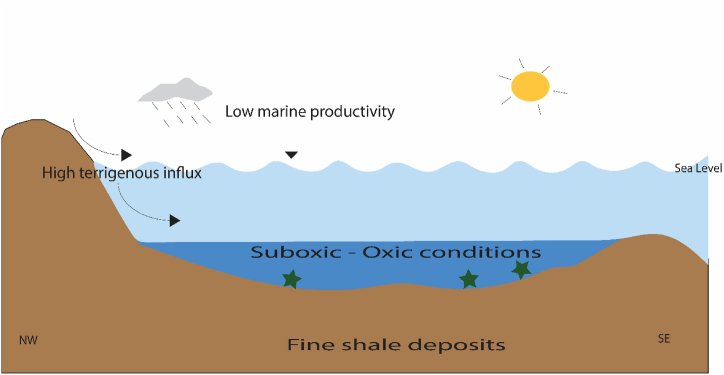
A model showing the factors controlling organic matter distribution in the Dohol Formation.

The proposed model suggests that the trends in organic matter accumulation and preservation in the Dohol Formation was affected by paleoclimate conditions, low marine productivity, oxidized conditions, and dilution effects caused by detrital input (Fig. 15). The shales were formed in a marine environment with abundant oxygen, which prevented organic matter from being preserved in the sediments (Fig. 15). Within the depositional environment, an increased influx of sediments occurred, resulting in a reduction of the organic matter concentration. Consequently, there was a decrease in the levels of organic matter and a reduction in the preservation of organic matter. The shales have limited organic content due to the low marine productivity (Fig. 15). Inadequate marine productivity can lead to a lack of organic matter due to the fact that the majority of organic matter in the ocean is produced by phytoplankton through photosynthesis, with only a little fraction being carried to the deep ocean [80]. The majority of the organic matter produced by phytoplankton is metabolised and transformed back into dissolved inorganic compounds within the upper layer of the ocean [80]. This process allows for the recycling of organic matter, making it available for use by phytoplankton. The ocean's productivity is constrained by the presence of nutrients, specifically nitrogen and phosphorus, which are vital for the proliferation of phytoplankton [81]. Oceanic water has a relatively low mineral concentration compared to sediments in terrestrial ecosystems, which limits the growth of phytoplankton. Therefore, a deficiency in marine productivity could lead to a shortage of organic matter in the ocean.

## 6 Conclusions

The following conclusions were drawn from the geochemical and mineralogical analysis of this study. The origin of the shales was identified as felsic igneous, implying a significant presence of feldspar, quartz, and elements including Si, O, Al, Na, and K. This deduction was further validated through investigation using SEM-EDX. It was determined that the shales from the Dohol Formation are from a passive margin setting, which suggests their formation in an area of low tectonic activity. The research indicates that the shales originate from a warm and humid climate. The analysis of paleoweathering conditions indicates that the shales have seen a significant degree of weathering, potentially accounting for the observed low elemental composition of the shales. The organic enrichment analysis indicates that the shales have low levels of organic matter due to a combination of factors, including a significant influx of terrigenous material, low marine productivity, and the presence of an environment with oxygen levels ranging from oxic to suboxic during sediment deposition.

## Funding statement

The authors express their gratitude to 10.13039/501100005710Universiti Teknologi PETRONAS for providing funding to this research under the 10.13039/501100016152YUTP Grant (015LC0-363).

## Data availability statement

Data will be provided upon request.

## CRediT authorship contribution statement

**Alidu Rashid:** Writing – review & editing, Writing – original draft, Methodology, Formal analysis, Data curation, Conceptualization. **Numair Ahmed Siddiqui:** Writing – review & editing, Supervision, Resources, Project administration, Funding acquisition. **Nisar Ahmed:** Writing – review & editing, Methodology, Conceptualization. **Ali Wahid:** Writing – review & editing, Formal analysis. **Muhammad Jamil:** Writing – review & editing, Formal analysis. **Abdul Aziz Sankoh:** Writing – review & editing, Formal analysis. **John Oluwadamilola Olutoki:** Writing – review & editing, Formal analysis.

## Declaration of competing interest

The authors declare that they have no known competing financial interests or personal relationships that could have appeared to influence the work reported in this paper.

## Figures and Tables

**Table 1A:** Data of some calculations used for the plots.

Sample	Formation	Eu/Eu*	Ce/Ce*	DF1	DF2	LREE/HREE	CIA*	PIA*	CIW	WIP	K2O/Al2O3	SiO2/Al2O3	Al2O3/TiO2	Na2O + CaO
S1	Dohol	1.22	2.51	0.04	8.45	16.19	65.8	91	105.04	98.14	0.52	2.17	12.61	0.02
S2	Dohol	0.77	0.93	0.49	3.25	8.53	72.8	91	106.08	65.31	0.40	3.15	12.43	0.02
S3	Dohol	0.63	0.95	−13.07	13.64	7.19	56.7	107	112.89	114.67	0.81	3.50	3280.00	0.01
S4	Dohol	0.53	0.95	−5.07	−2.80	6.83	97.7	101	116.32	16.48	0.15	6.73	15.34	0.01
S5	Dohol	0.72	1.70	−7.52	4.32	13.77	77.3	102	110.49	58.51	0.36	3.69	13.72	0.00
S6	Dohol	0.52	0.91	−12.69	8.51	7.13	66.7	103	114.08	85.91	0.58	3.74	8.47	0.01
S7	Dohol	0.58	1.14	−7.56	4.11	8.68	69.9	100	115.20	61.41	0.52	5.15	25.00	0.01
S8	Dohol	0.57	1.21	−4.52	2.69	9.20	77.8	101	112.20	50.83	0.36	4.25	54.12	0.01
S9	Dohol	0.63	0.94	−2.64	2.88	7.51	78.3	100	111.23	55.25	0.35	3.62	52.36	0.01
S10	Dohol	0.59	1.09	−4.07	4.16	8.34	78.0	100	108.71	57.60	0.33	3.38	47.32	0.01
S11	Dohol	0.71	1.16	−7.73	4.40	9.01	73.3	100	113.17	59.23	0.44	4.61	32.24	0.01
S12	Dohol	0.69	1.16	−4.99	3.45	8.76	76.4	101	113.38	56.07	0.39	4.12	39.53	0.01

**Table 2A:** Data of some calculations used for the plots.

Sample	Formation	Sc/Cr	La/Y	La/Sc	Y/Ni	Cr/V	La/Th	Zr/Sc	Th/Sc	Zr/Hf	Rb/Sr	Th/U	Co/Th	La/Co	Th/Co	Th/Cr
S1	Dohol	0.69	3.16	1.02	1.11	0.26	0.59	8.31	1.73	5.11	5.80	10.85	0.03	17.86	30.11	1.19
S2	Dohol	1.15	2.97	0.76	0.27	0.12	0.59	4.52	1.30	20.58	6.47	16.33	0.02	26.83	45.61	1.50
S3	Dohol	0.78	2.47	0.51	0.21	0.21	0.26	6.46	1.93	29.70	9.38	16.86	0.01	22.01	83.31	1.51
S4	Dohol	0.51	3.57	3.24	1.21	0.28	0.53	13.33	6.11	1.11	5.63	10.71	0.14	3.68	6.94	3.09
S5	Dohol	1.28	3.04	1.20	1.44	0.39	0.75	8.44	1.59	26.53	13.14	11.22	0.04	21.47	28.52	2.04
S6	Dohol	1.22	1.70	0.58	3.09	0.40	0.65	4.73	0.89	23.93	7.85	15.78	0.06	10.08	15.40	1.09
S7	Dohol	1.88	3.21	0.79	1.75	0.35	0.78	3.99	1.01	8.91	8.34	12.38	0.07	11.25	14.50	1.90
S8	Dohol	0.99	2.86	0.97	0.93	0.27	0.58	6.55	1.68	11.01	7.77	13.21	0.04	14.05	24.34	1.66
S9	Dohol	1.56	2.97	0.54	0.41	0.19	0.44	3.61	1.23	8.80	6.99	14.34	0.03	12.95	29.77	1.91
S10	Dohol	1.71	3.31	0.69	1.03	0.31	0.65	3.98	1.05	12.04	8.59	11.70	0.04	14.65	22.45	1.80
S11	Dohol	1.22	3.65	0.99	0.86	0.26	1.05	5.24	0.94	10.86	7.76	8.90	0.06	17.52	16.66	1.15
S12	Dohol	1.18	2.94	0.82	0.86	0.26	0.59	5.42	1.39	10.86	7.76	10.53	0.04	14.11	23.77	1.64
